# Selective inhibitors of the PSEN1–gamma-secretase complex

**DOI:** 10.1016/j.jbc.2023.104794

**Published:** 2023-05-09

**Authors:** Lutgarde Serneels, Rajeshwar Narlawar, Laura Perez-Benito, Marti Municoy, Victor Guallar, Dries T’Syen, Maarten Dewilde, François Bischoff, Erwin Fraiponts, Gary Tresadern, Peter W.M. Roevens, Harrie J.M. Gijsen, Bart De Strooper

**Affiliations:** 1 Department of Neuroscience, KU Leuven, Leuven, Belgium; 2 Center for Brain and Disease Research, VIB, Leuven, Belgium; 3 Deparment of Discovery Chemistry, Janssen Research & Development, Janssen Pharmaceutica NV, Beerse, Belgium; 4 Deparment of Computational Chemistry, Janssen Research & Development, Janssen Pharmaceutica NV, Beerse, Belgium; 5 Nostrum Biodiscovery, Barcelona, Spain; 6 Life Sciences Department, Barcelona Supercomputing Center, Barcelona, Spain; 7 Institució Catalana de Recerca i Estudis Avançats (ICREA), Barcelona, Spain; 8 Charles River Laboratories, Beerse, Belgium; 9 Campus Strategy & Partnerships, Janssen Pharmaceutica NV, Beerse, Belgium; 10 Dementia Research Institute, University College London, London, UK

**Keywords:** ƴ-secretase, inhibitors, selectivity, therapy, medicinal chemistry

## Abstract

Clinical development of γ-secretases, a family of intramembrane cleaving proteases, as therapeutic targets for a variety of disorders including cancer and Alzheimer’s disease was aborted because of serious mechanism-based side effects in the phase III trials of unselective inhibitors. Selective inhibition of specific γ-secretase complexes, containing either PSEN1 or PSEN2 as the catalytic subunit and APH1A or APH1B as supporting subunits, does provide a feasible therapeutic window in preclinical models of these disorders. We explore here the pharmacophoric features required for PSEN1 *versus* PSEN2 selective inhibition. We synthesized a series of brain penetrant 2-azabicyclo[2,2,2]octane sulfonamides and identified a compound with low nanomolar potency and high selectivity (>250-fold) toward the PSEN1–APH1B subcomplex *versus* PSEN2 subcomplexes. We used modeling and site-directed mutagenesis to identify critical amino acids along the entry part of this inhibitor into the catalytic site of PSEN1. Specific targeting one of the different γ-secretase complexes might provide safer drugs in the future.

The γ-secretases are fascinating membrane-bound protease complexes with great potential for therapeutic applications in Alzheimer’s disease (AD) (1, 2, 3), cancer (4, 5, 6, 7), acoustic trauma (8, 9), peritoneal fibrosis (10), and atherosclerosis (10, 11). Their role in AD is of particular interest because dominant inherited mutations in the catalytic subunits (presenilin (PSEN)1 and PSEN2) of these enzymes are sufficient to cause the full neuropathological and clinical spectrum of this brain disorder. Indiscriminate inhibition of all the γ-secretases with “broad” spectrum γ-secretase inhibitors (GSIs) causes, however, severe mechanism-based side effects. This was very well exemplified by the side effects of semagacestat, a broad-spectrum inhibitor, in a phase III clinical trial for AD (3).

One of the problems is the pivotal function of γ-secretase processing in Notch (6) signaling which maintains tissue homeostasis, especially of gut, immune system, and skin. However, besides Notch and Amyloid beta Precursor Protein (APP), more than a hundred other substrates for γ-secretases have been identified (12), making it difficult to interpret the side effects only in terms of Notch inhibition. It seems crucial to develop more targeted and specific approaches to modulate these enzymatic activities.

We have previously argued that not only the lack of selectivity but also the pharmacodynamic properties of semagacestat have strongly contributed to the side effects (2). Because of the short half-life of the inhibitor, Notch signaling was intermittently but very effectively blocked, thus enhancing Notch side effects, while the “area under the curve” for the inhibition of Aβ peptide was minimized, resulting in lack of effect on the target (2). Recent breakthroughs by Yigong Shi *et al.* have delivered cryo-EM structures of the γ-secretases bound to APP (13), Notch (14), or to different inhibitors (15) and open new interesting avenues toward specific modulation of these enzymes and their substrates. Very promising is the identification of a γ-secretase allosteric site (15) which when bound by the small modulator E2021 drives the processing of APP toward shorter Aβ fragments. Targeting this allosteric site could be combined with targeting the binding site of APP, which might increase efficiency and specificity of such inhibitor (15). The cryo-EM structures of γ-secretase with APP (13) and Notch substrates (14) also indicate additional space and flexibility in their binding sites. It might be possible to exploit this to generate inhibitors that increase selectivity for APP (in AD) or for Notch (in cancer), but it is unclear to what extent other important substrates of γ-secretase could be spared. It will remain crucial to dose carefully any novel drug to determine whether a therapeutic window can be established.

A third approach is the generation of inhibitors specific for one of the different γ-secretase complexes. The best-known example of such complex-specific inhibitor is MRK-560 (16, 17). A recent structure was published showing that MRK-560 binds PSEN1 but not PSEN2 (18), providing the molecular basis for its selectivity. MRK-560 is a cyclohexyl sulfone derivative (16, 17) and lowers Aβ production without causing any Notch-related side effects in mice (19). Remarkably, this compound does not show selectivity *versus* APP or Notch processing *in vitro* (16, 17) but displays, like another selective GSI SCH-1500022, high selectivity toward the PSEN1–γ-secretase complex (37- and 250-fold, respectively) compared to PSEN2–γ-secretase complexes (20). The exceptional and differential effects of MRK-560 on Notch and APP processing *in vivo* were further explored in WT and PSEN2-deficient mice. MRK-560 potently and dose dependently reduced Aβ levels in both models (19), but while MRK-560 treatment in WT mice was safe, treatment of mice genetically deficient for PSEN2 caused major Notch-related toxicity. This experiment demonstrates that PSEN2 complexes can take over a large part of Notch processing in peripheral organs when PSEN1 complexes are pharmacologically inhibited. The effect on other substrates was not further investigated, but overall, the mice looked healthy, suggesting a reasonable therapeutic window for this compound also *versus* other known and unknown substrates of the γ-secretases. A follow-up study using MRK-560 for the treatment of T cell acute lymphoblastic leukemia (7) confirmed that selective inhibition of PSEN1 complexes in the cancer cells protected the mice, while side effects in gut and skin (protected by residual PSEN2 activity) were not observed, further extending the concept that selective inhibition of γ-secretase complexes might be a fruitful avenue toward therapeutic applications. Additional evidence that the heterogeneity of γ-secretase is worthwhile to explore comes from genetic experiments in which the APH1B subunit was selectively deleted. In this model, Aβ plaque formation and memory problems in an AD mouse model were rescued, while Notch signaling overall seemed unaltered (21). This has led us to speculate that an inhibitor with maximal selectivity for PSEN1 over PSEN2 and, if possible, APH1B over APH1A complex would be preferred for further exploration for the treatment of AD.

These preclinical observations warrant the further generation of effective γ-secretase complex–specific inhibitors, and we set out here to define the pharmacophoric criteria for such a selective inhibitor. γ-Secretase complexes consist of four essential proteins, that is, PSEN, nicastrin (NCSTN), anterior pharynx defective 1 (APH1), and presenilin enhancer 2 (PSENEN) (22, 23, 24, 25, 26). PSEN harbors the aspartyl catalytic core (27) but becomes only active when the three other subunits are associated in a 1:1:1:1 stoichiometry. The assembly occurs during the trafficking of the protein from the endoplasmic reticulum to the cell surface and involves proteolytic and conformational maturation changes (28). Two PSEN proteins (PSEN1 and PSEN2) and two APH1 proteins (APH1A and APH1B) are encoded by separate genes (26). PSEN1 and PSEN2 differ in 35% of their sequence, while APH1A and APH1B are 44% different (29). Alternative splice variants of the transcripts of these genes exist, and posttranslational modifications, lipids and proteins modify the activity of the enzymes.

Here, we focus on the four major γ-secretase complexes (PSEN1–APH1A, PSEN1–APH1B, PSEN2–APH1A, and PSEN2–APH1B). We have generated four cell lines that each express only one of these four major forms of γ-secretase and use those to investigate the pharmacophoric properties of different, previously generated, GSIs. Based on the rational design, a novel aza-bicyclooctane sulfonamide was synthesized with low nanomolar potency toward PSEN1–APH1B complex and high selectivity *versus* PSEN2-APH1A and PSEN2-APH1B.

## Results

### A novel cellular assay to measure the activity of the four individual γ-secretase complexes

Mouse embryonic fibroblast (MEF) cells deficient for the *Psen* and *Aph1* genes (*Psen1/2*^−/−^, *Aph1ABC*^−/−^) were reconstituted with human *PSEN1* or *PSEN2* and with human *APH1A* or *APH1B* to generate four independent cell lines, each constitutively expressing exclusively one type of γ-secretase complex (Fig. S1*A*) (19). A human APP-C99-GFP reporter was introduced to enable the measurement of Aβ peptides in the conditioned media of the cells to monitor γ-secretase activity. The assay was established in a 96-well format. The four cell lines typically secreted between 25 and 250 pg/ml Aβ peptides per hour. As shown in Fig. S1*B* and Table 1, the transition state analog inhibitor (TSAI) L-685,458 (30) inhibited all the four complexes within similar ranges (95% CI: PSEN1-APH1A: 1206–2366 nM, PSEN1-APH1B: 597–3862 nM, PSEN2-APH1A: 992–2595 nM, PSEN2-APH1B: 2220–5737 nM). We benchmarked the assay with the PSEN1 selective inhibitor MRK-560 (31), confirming that this compound is 100- to 350-fold more potent in inhibiting PSEN1 complexes (low nM range) than PSEN2 complexes (>130 nM) (Table 1 and Fig. S1).

**Table 1:** Inhibitory activity data for known GSIs towards specific γ-secretase complexes




The means (bold) of the number of experiments is indicated (N), and 95% CI are indicated between brackets. GraphPad Prism 7 software was used to generate inhibition fitting curves (four-parameter logistic equation, nonlinear regression) and to determine IC50 and 95% CI values (Fig. S2). Selectivity values (IC50 ratios of PSEN1 *versus* PSEN2) above 10 are highlighted in red. Experiments were performed with the same cell lines at either Janssen (B) or KU Leuven (L) depending on compound availability, and five compounds were tested at both sites and showed agreement in selectivity although the IC50 is different at the two sites, see Fig. S2.

### Selectivity profile of known GSIs

Multiple classes of small-molecule GSIs have been reported. We selected compounds covering most of the known chemical and functional classes from previously published work (15, 32) (Table 1). These inhibitors have been classified as TSAIs, allosteric nonselective inhibitors, “Notch sparing” inhibitors, “PSEN1-selective” inhibitors, and “Notch sparing PSEN1-selective” inhibitors (33, 34). These names should probably be revised taking into account more recent understanding of the binding sites of these compounds (15). We tested the different inhibitors in the four cell lines. Several of the well-studied GSIs (L-685,458, TSAI-1, LY411575, semagacestat, RO-4929097, DAPT, and DAPT analog) display <10-fold selectivity toward the different γ-secretase complexes and we call them therefore “broad-spectrum” inhibitors. Other compounds (entries 8–12) show moderate (between 10- and 100-fold) to high (above 100-fold) selectivity for PSEN1 complexes *versus* PSEN2 complexes. The reverse selectivity (PSEN2 > PSEN1) was not seen, likely because no systematic screens were performed to identify PSEN2 selective compounds. MRK-560 is the prototype of a PSEN1 selective inhibitor and stands out in terms of potency and selectivity. In previous publications, MRK-560 was shown in cell-free *in vitro* assays to have a 37-fold (20) or a 5-fold (18) selectivity for PSEN1 complexes *versus* PSEN2 complexes (22). We confirm here in our cell-based assay nanomolar potency toward PSEN1-APH1B (0.42 nM, 95% CI: 0.39–0.45 nM), PSEN1-APH1A (1.4 nM, 95% CI: 1.3–1.5 nM), and >100-fold selectivity *versus* PSEN2 complexes (PSEN2–APH1A and PSEN2–APH1B). From Table 1, it is clear that some of the other GSIs show also selectivity toward PSEN1 complexes, but this was not achieved by a rational drug design and was not documented before in a systematic way. As the Notch sparing activity of MRK-560 appears largely explained by its PSEN1 selectivity, we investigated the complex selectivity of compounds claimed to have some Notch-sparing effect in previous clinical studies. Interestingly, begacestat (35) and avagacestat (36) display moderate selectivity (<41) for PSEN1 complexes *versus* PSEN2 complexes. While this selectivity might potentially lower side effects in the clinic (as predicted based on preclinical work with MRK-560 (7, 16, 19)), it is unclear whether these compounds were tested at doses that exploited this moderate selectivity. In any event, clinical development of those two compounds was halted prematurely because of similar side effects (37) as observed with the broad-spectrum inhibitor semagacestat (3).

### Towards PSEN1-complex selective GSIs

Yang *et al.* (15) reported an avagacestat-bound cryo-EM structure of human γ-secretase. We were particularly interested to understand how avagacestat binds to the γ-secretase complex as it displays a moderate selectivity *versus* PSEN2 complexes in our hands. Contrary to previous observations (38), the EM structure indicates that GSI-binding residues are located in the pocket that is formed by TM1-2, TM6-9, and β strands but not TM3-5 of PSEN1 (15). While the conformation of TM6a and the PAL motif in the semagacestat and the L-685,458-bound γ-secretase structures are nearly identical to the substrate-bound (APP and Notch) states, avagacestat induces a change in the TM6a and the PAL motif, rotating these structures and moving them away from the active site. The more recent cryo-EM structure of MRK-560 bound to PSEN1- and PSEN2-human γ-secretase (18) confirmed that MRK-560 binds the same site as avagacestat and that in particular, two amino acids T281 and L282 located in the hydrophobic region of loop 6 are critical for PSEN1 selectivity of this compound. Unlike semagacestat and avagacestat, MRK-560 forms H-bonds with N385, L282, and L432 of PSEN1, and the sulfonamide group is much closer to loop-2. Avagacestat forms only one H-bond with G382 of PSEN1, and the substituents are a bit farther from loop-2 and do not make any interaction. It was hypothesized that these additional interactions with loop-2 by MRK-560 drive the isoform selectivity.

At the time when this work was executed, the available cryo-EM structures of the PSEN1–γ-secretase complex had a relatively low resolution of 4.2 Å (39, 40), and we therefore applied ligand-based design approaches, comparing the structures of the GSI molecules to elucidate common features of potent and complex selective GSIs. The cyclohexyl sulfone derivative (Fig. S2, *C* and *D*), a MRK-560 analog without the trifluoromethyl sulfonamide functionality, displayed almost 60-fold less potency for PSEN1–APH1B (25.8 nM [95% CI: 17.0–73.4 nM]) and a significant drop in selectivity *versus* PSEN2 complexes (11–41-fold), suggesting the importance of the sulfonamide group and the presence of the H-bond donor to increase both potency and selectivity. MK-0752, propagated as a clinical candidate for oncology indications (41), displays low nanomolar potency toward PSEN1–APH1A and PSEN1–APH1B (5.3 nM [95% CI: 2.3–10.3] and 1.6 nM [95% CI: 0.8–2.5], respectively) but moderate selectivity *versus* PSEN2–APH1A and PSEN2–APH1B complexes (14- and 70-fold, respectively). ELN475516 (34), reported as a Notch-sparing inhibitor, displayed decent potency (10.3 nM [95% CI: 9.1–11.5]) for the PSEN1–APH1B complex and moderate selectivity (37-fold) *versus* PSEN2 complexes. A follow-up compound of the same series ELN318463 was also selected for its Notch-sparing effect and PSEN1 selectivity (34, 38). ELN-318463 is equipotent to ELN475516 toward the PSEN1–APH1B complex (24.5 nM [95% CI: 18.1–32.0]) but displays higher selectivity *versus* PSEN2 complexes (70-fold).

**Figure 1:**
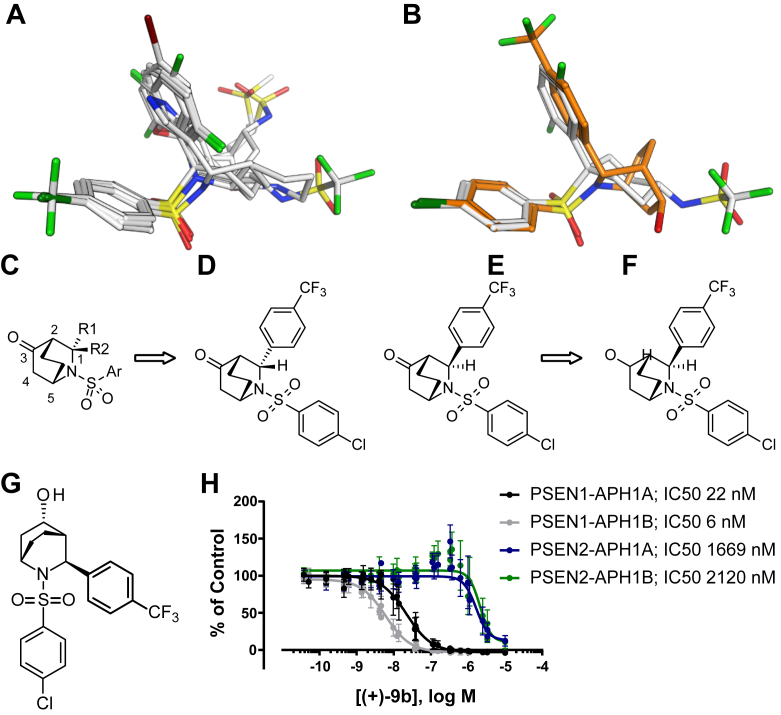
**Overlay of ELN318463, ELN475516, SCH-900229, SCH-1500022, and MRK-560.***A*, the flexible alignment of known GSI using the crystal structure of compound ELN318463 (CCDC 764935) as reference. *B*, the overlay of the designed inhibitor (in *orange*) with MRK-560. Aryl substitution at “1” position of bicyclic core (*C*) results in *endo* (*D*) and *exo* (*E*) isomers. Computational modeling studies indicated that the *exo* isomer would provide us the desired “U” conformation between the 4-chlorophenyl sulfonamide and 4-trifluromethyl phenyl moieties. *F*, hydroxy group at position “3” of the bicyclic core provides an H-bond donor as in MRK-560. *G*, chemical structure of (+)-9b. *H*, dose-dependent effect of compound (+)-9b on Aβ_40_ peptides generated by the MEF cells expressing the different ƴ secretases. The data shown are means of >46 experiments. GraphPad Prism 7 software was used to generate inhibition fitting curves (four-parameter logistic equation, nonlinear regression) and to determine IC50 values and 95% CI. Fits to the Hill equation yield IC50 values of 22 nM (20–24 nM, 95% CI), 6 nM (5.8–6.4 nM, 95% CI), 1669 nM (1471–1883 nM, 95% CI), and 2120 nM (189–2352 nM, 95% CI) for PSEN1-APH1A, PSEN1-APH1B, PSEN2-APH1A, and PSEN2-APH1B, respectively. APH1, anterior pharynx defective 1; GSI, γ-secretase inhibitor; MEF, mouse embryonic fibroblast; PSEN, presenilin.

Small-molecule X-ray crystal structures of the MRK-560 analog (42) and of ELN47551635 (43) have been solved and reveal a unique “U” conformation between 4-chlorophenyl sulfone/sulfonamide and 2,5-fluorophenyl/pyrazole moieties (Fig. 1). While the ligand X-ray conformation shown here may not be equivalent to the bioactive conformation, the relative lack of conformational freedom of these molecules suggests that this low-energy conformation is particularly favorable.

We applied the knowledge summarized in Table 1 to identify pharmacophoric features required for PSEN1–PSEN2 complex selective inhibition. We selected ELN-318463, ELN-475516, and MRK-560. We also included SCH-900229 (44), a fused bicyclic GSI, that was reported to be PSEN1-selective with 20-fold selectivity *versus* PSEN2 and SCH-1500022 (20), a fused tricyclic GSI, with a reported 250-fold selectivity toward PSEN1 *versus* PSEN2, with low nanomolar potency. We aligned ELN-318463, ELN-475516, SCH-900229, SCH-1500022, and MRK-560 to the reference crystal structure of ELN-47551635 (CCDC 764935) (43) using the Molecular Operating Environment (MOE) software flexible alignment tool (https://www.chemcomp.com/Research-Citing_MOE.htm). The overlay of the ligands superposes the common aryl-sulfone or sulfonamide motif present in all the molecules displayed in Figure 1*A*. The molecules adopt a “U” conformation where two of the aromatic rings of each molecule form intramolecular stacking interactions, aligning with the ligand X-ray structures. The overlay shows well-conserved matching of the two aromatic centers. One aromatic ring is often substituted with small hydrophobic groups such as Cl and CF_3_ in the para position, while the other tolerates more structural variation and is often substituted in several positions with groups such as F, Br, and CF_3_. The third branch of the molecules, although displaying more structural diversity, also shows considerable overlap. In the center of the molecule, a saturated cycle is allowed, while further substituents in the third branch include more polar groups such as sulfonamide (Fig. 1*A*). Given the similarity of MRK-560 with cyclohexyl sulfone, we hypothesized that this third branch may be the origin of the improved selectivity of MRK-560 toward PSEN1 *versus* PSEN2 complexes.

Based on these observations, we explored several bicyclic and tricyclic amine scaffolds that could lead us to the same desired 3D arrangement of functional groups. A [2,2,2] aza-bicyclooctanone (Fig. 1*C*) scaffold turned out to have a good cyclic core that can be substituted with aryl and particularly aryl sulfonamide groups to provide the putative vital “U” conformation. Any aryl substitution at “1” position of the bicyclic core results in *endo* and *exo* isomers (Fig. 1, *B*–*E*). Computational modeling studies indicated that the *exo* isomer would provide us the desired “U” conformation between the 4-chlorophenyl sulfonamide and 4-trifluromethyl phenyl moieties as depicted in Figure 1*B*. Moreover, the ketone group at position “3” of the bicyclic core could be used as a handle to install various functionalities and understand their impact on potency and selectivity (Fig. 1*F*). We synthesized a series of azabicyclo[2.2.2]octane sulfonamides, which will be the subject of a separate publication. Introducing an H-bond donor *via* an OH alcohol in the position of the branched substituent matched the pharmacophore, and we speculated that this might satisfy the features necessary for improved selectivity. Indeed, compound (+)-9b (Fig. 1*G*) appeared to be a very potent PSEN1–APH1B complex–selective GSI (IC_50_ of 6 nM [95% CI: 5.8–6.4]) with moderate selectivity *versus* PSEN1–APH1A (IC_50_ of 22 nM [95% CI: 20–24 nM]) and >250-fold selective for PSEN1 *versus* PSEN2 (Fig. 1*H*).

### Structural determinants of γ-secretase selective inhibition

We performed computational simulations to elucidate the binding trajectory and site of compound (+)-9b into the cryo-EM γ-secretase structure. All-atom Protein Energy Landscape Exploration (PELE) (45) Monte Carlo simulations were performed. PELE recapitulates binding trajectories and poses for diverse protein ligand receptor systems (46, 47, 48) including membrane proteins such as G protein-coupled receptors (49). The stability of the binding poses was investigated using molecular dynamics (MD) simulations.

**Figure 2:**
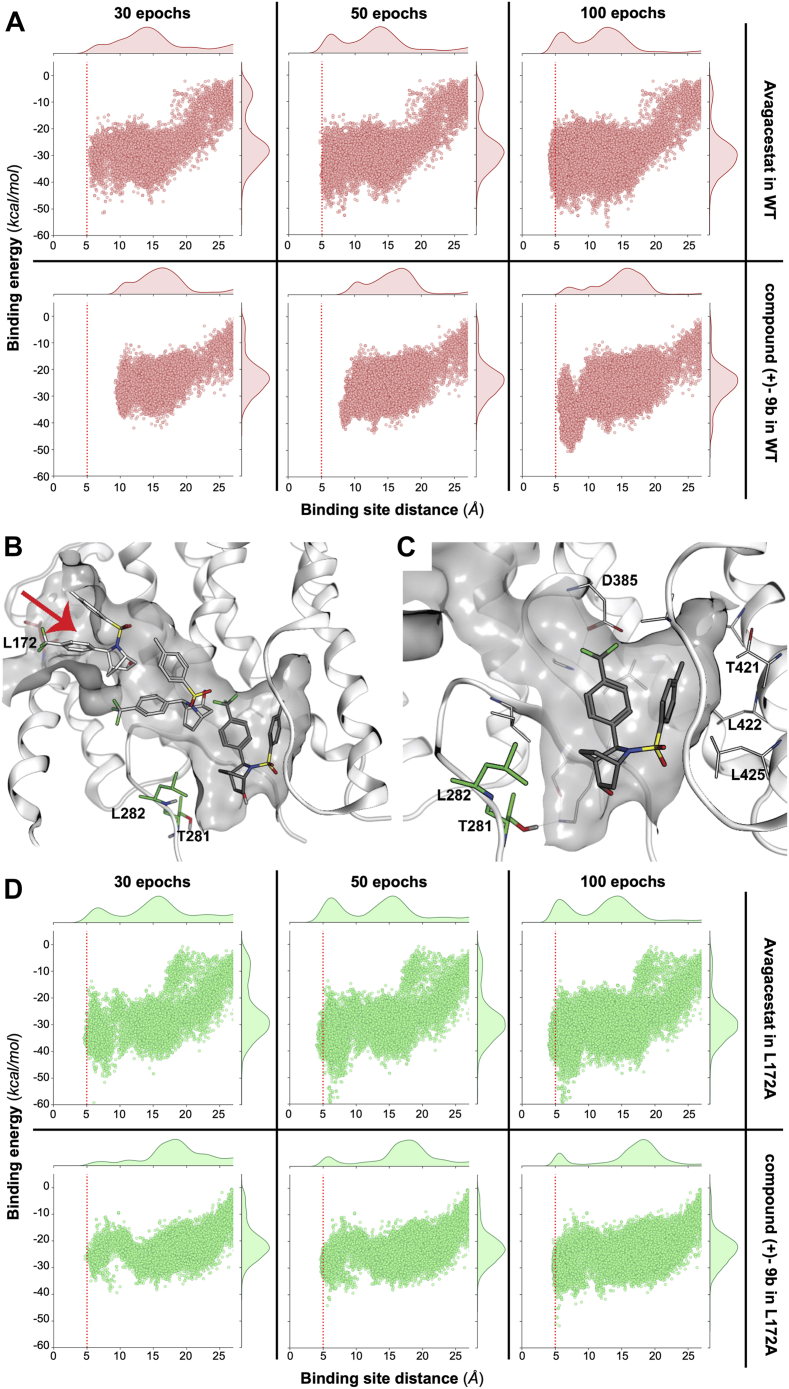
**Energetic and structural PELE results.***A*, the energetic profiles obtained at different stages (epochs) of the simulation for the binding of avagacestat and compound (+)-9b, first and second row, respectively, in the WT receptor (15). A higher number of epochs means running more MC (Monte Carlo) steps and increasing the effort to achieve the bound conformation, represented with a vertical *red line* at a distance of 5 Å to the binding site center. *B*, a view of the initial ligand close to L172 (in *red*) at the entrance of the channel, capturing different snapshots of compound (+)-9b from the MC simulations, reaching the final site as seen in the new structure (PDB ID 7Y5T) (18) of MRK-560, in *green sticks* L281 and T282. *C*, a closed-up view of the binding site for compound (+)-9b (*gray sticks*) with some important amino acids involved in the interactions (*D*). Equivalently to panel *A*, the energetic profiles are displayed at different stages (epochs) of the simulation of avagacestat and compound (+)-9b in the mutated L172A receptor.

In more detail, we performed “out-in” Monte Carlo simulations using PELE version 1.6.1 (see Experimental procedures sections and videos S1 and S2). We first studied the binding of both compound (+)-9b and avagacestat with WT γ-secretase. All systems and simulations were prepared and performed in the same way using the cryo-EM structure PDB ID 6LQG (15) with crystallographic ligands removed and the ligands under study placed randomly outside the receptor. The trajectory of the binding simulation was analyzed by comparing the binding energy between the ligand and receptor, with the distance from the putative binding site seen in the 6LQG structure. Figure 2*A* shows the energetic profiles obtained at different stages (epochs) of the simulation for the binding of avagacestat and compound (+)-9b in the PSEN1 WT receptor. Both ligands can reach the binding site by the last epoch of the simulation, although recapitulation of avagacestat binding delivers a higher density of poses in the binding site location. Interestingly, the lowest binding energy conformations for compound (+)-9b clearly correspond to binding poses in the anticipated site.

The simulations allowed us to visualize the pathway for the ligand into the binding site (Fig. 2*B*). Interestingly, the ligands traverse a narrow channel that begins at leucine 172 before finally binding in a site on the intracellular side of the receptor (Fig. 2*C*). We analyzed the binding pose with respect to amino acids that differ between PSEN1 and PSEN2, with the expectation that the high selectivity for PSEN1 would involve interaction with nonconserved amino acids. Overall, the PSEN1 and PSEN2 sequences are highly conserved (∼75% sequence similarity and ∼65% sequence identity), but T281 and L282 have emerged as important determinants for PSEN1 *versus* PSEN2 selectivity (18). Both are located in a flexible loop and observed to interact with the ligand (Fig. 2*C*). Other reports have suggested that L172 is also important for the selectivity of certain GSIs, especially those including the sulfone group (38). However, this amino acid is far from the binding site, approximately 20 Å, but is located at the entrance of the binding channel. This led us to hypothesize that the channel itself could play a role in selectivity.

**Figure 3:**
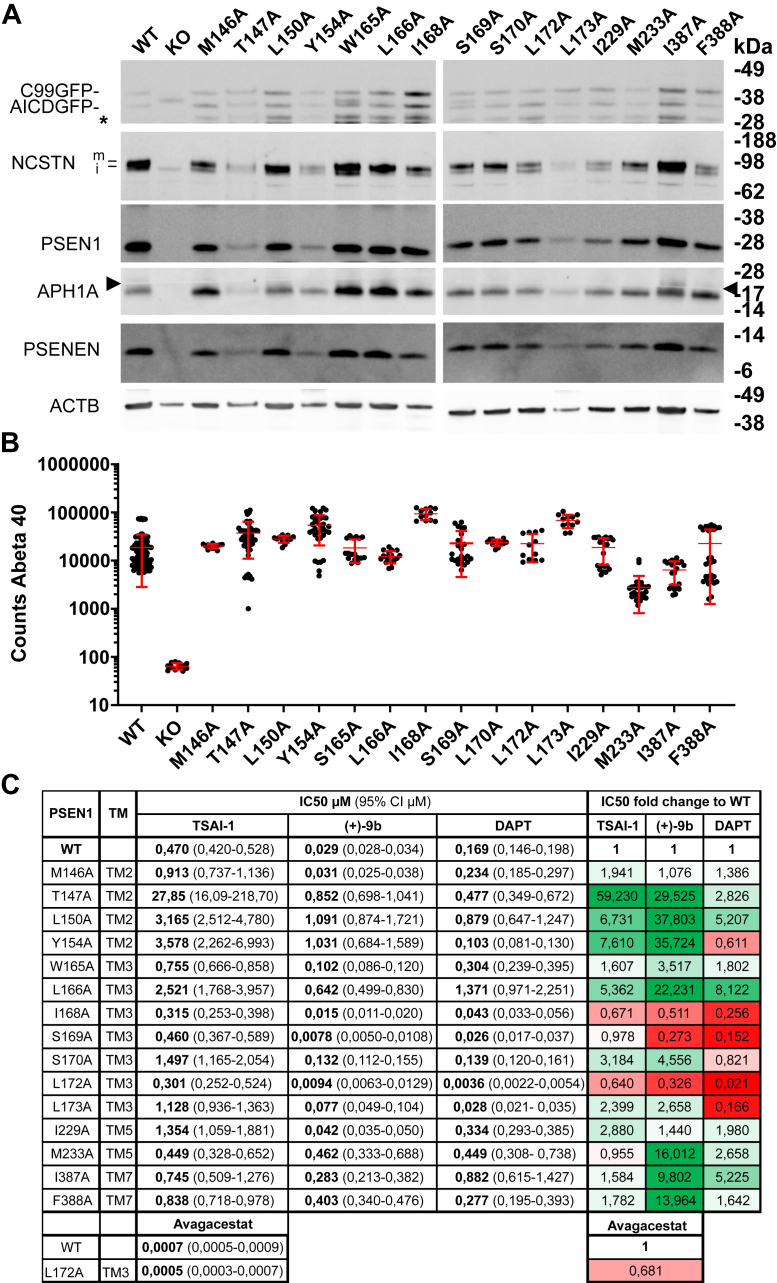
**γ-Secretase activity and IC**_**50**_**values for three GSI’s tested in the single alanine mutated PSEN1–AH1A complexes-restored cell lines.***A*, Western blot analysis of mixed pools of transfected PSEN knockout fibroblasts analyzing the protein levels of NCSTN, PSEN1, APH1A, PSENEN, C99-GFP reporter. ACTB is the loading control. Maturation of NCSTN (upper band indicated with m), stabilization of PSENEN, and generation of AICD-GFP and Aβ40 demonstrate that the mutated PSEN1 are incorporated into the γ-secretase complex. A blot of the KO cell lysate is shown as control. Immature NCSTN (lower band indicated with i) is migrating faster, PSEN1 and APH1A are absent, PSENEN is unstable and the C99-GFP is converted to C83-GFP by α-secretase activity, and Aβ40 secretion is absent, all as expected. Molecular size markers are indicated, ∗ is an unspecific band, and the *arrowheads* indicate a fusion line of the blots. *B*, sandwich ELISA of secreted Aβ40 in the media from the cell pools transfected with the different mutants demonstrate that all mutants support γ-secretase activity, mean ± SD is indicated in *red*. This measurement is taken as the 100% activity in each cell pool. *C*, IC50 values (μM) for Aβ_40_ secretion for the indicated GSI and mutant PSEN1 as measured in a dose-response curve. The later panel displays relative changes compared to the WT coded by color code (*green* is an increased IC50 value indicative for a lower binding affinity, *red* is a reduced IC_50_ indicative for a higher binding affinity). *N* = 3 to 4 experiments, data are presented as mean with 95% CI given between brackets. APH1, anterior pharynx defective 1; GSI, γ-secretase inhibitor; NCSTN, nicastrin; PSEN, presenilin; PSENEN, presenilin enhancer.

We turned to experimental site–directed mutagenesis and selected a series of amino acids (Fig. 3) in the entrance channel for mutation to alanine. We determined the effect of these mutants on PSEN1–APH1A activity and the consequences of inhibiting γ-secretase activity by the nonselective TSAI-1, DAPT, and the PSEN1 complex-specific (+)-9b inhibitor (Figs. 3 and S3). Cell pools were generated to restore PSEN1–APH1A γ-secretase with the indicated PSEN1 alanine mutants as shown in Figure 3. While the absolute levels of the expression of the different mutants were variable as we did not perform clonal selections, the mutant cell pools all expressed reconstituted γ-secretase complex as demonstrated by the maturation of NCSTN and stabilization of PSENEN (Fig. 3*A*). More importantly, they were all enzymatically active as shown by the secretion of Aβ40 in the supernatants of the cell cultures (Fig. 3*B*). The level of activity (which varies between cell lines, notice that this is a log scale) was set as 100%, and cells were treated with increasing concentrations of TSAI-1, (+)-9b, DAPT, and avagacestat to determine IC50.

As shown in Figure 3*C*, mutations of T147A, L150A, Y154A, L166A, and S170A decreased the binding affinity for compound TSAI-1. For compound (+)-9b, nine amino acid positions were found where alanine substitution affected its potency by decreasing its binding affinity. These amino acids reside in TM2 (T147A, L150A, and Y154A), TM3 (W165, L166A, and S170A), TM5 (M233), and TM7 (I387A and F388A). The mutation of the previously mentioned L172 as well as S169 to alanine caused an increase of the binding affinity for compound (+)-9b. For DAPT, the amino acids in TM2 (L150A), TM3 (L166A), and TM7 (I387A) caused a >3-fold decrease in binding affinity, while alanine substitution of the following four amino acids resulted in increased potency: I168A, S169A, L172A, and L173A. L172 is located in TM3, pointing toward the cell membrane. Alanine substitution of L172 showed an increase in binding affinity for all four tested ligands (TSAI-1, (+)-9b, DAPT, and avagacestat). For DAPT, this increase is strongest (47-fold) compared with 3.1-fold for (+)-9b and 1.5-fold for TSAI-1 and avagacestat.

The mutagenesis confirmed a role for L172 as a gatekeeper for entrance to the binding channel, along with the adjacent I168 and S169 amino acids. They are the only amino acids that when mutated to smaller alanine sidechains lead to an increase in activity of the GSIs, presumably allowing easier entrance to the channel. Mutation of other amino acids along the pathway do not increase activity, consistent with the channel entrance being the rate-limiting step. In fact, mutation of other amino acids is detrimental for activity, possibly suggesting the WT residues assist the ligand binding (expected given they are optimized lead compounds).

Finally, we returned to the computational simulations to examine the impact of the L172A mutation on the ligand-binding pathway and its energetic profile. All systems were prepared in the same way as previously described. Figure 2*D* shows the energetic profiles for the entrance of avagacestat and compound (+)-9b in the L172A variant. Both compounds showed a higher density of points at low binding site distances than WT, especially at low number of epochs. However, the impact of the L172A mutation is most notable for compound (+)-9b. After 50 epochs of simulation, the compound (+)-9b could reach the binding site only with the L172A variant (green dots). After more simulation time, at the 100th epoch, the ligand could be detected in the binding site of both WT and mutant. However, the density of points at low distances is significantly higher for the L172A variant. Overall, this is consistent with the L172A variant having a slightly wider entrance channel due to the replacement of leucine by alanine, allowing an easier ligand entrance. Thus, compound (+)-9b seems to be more affected by this mutation than avagacestat. This phenomenon may be because of the higher rigidity of compound (+)-9b due to the presence of a ring next to the sulfonamide group and fewer rotatable bonds.

## Discussion

Selective inhibition of one or more of the γ-secretase complexes specifically, instead of blocking all γ-secretases at once, gets traction from preclinical research observations (7, 21) as an alternative to the broad-spectrum inhibition of the enzymes. The latter approach has failed in phase III trials in the past because of side effects (3), although it has been argued that these experiments should be reassessed in the light of the bad kinetic properties of the drugs available at that time (2). Here, we investigated the structural requirements of compounds that selectively inhibit PSEN1–γ-secretase complexes *versus* PSEN2–γ-secretases as those were shown to provide a therapeutic window in preclinical models of AD and T-ALL (7, 19, 21).

We reassessed previously identified GSIs in a new cell-based assay that allows to measure the activity of the four different γ-secretase complexes separately (29). We show here that GSIs such as LY-411575, RO-4929097, and a DAPT analog are excellent inhibitors of all four γ-secretase activities with low nanomolar potencies, without convincing selectivity toward any of them. We call them “broad-spectrum” inhibitors. As expected, transition-state inhibitor analogs L-685,458 and TSAI-1 also display little or no selectivity. Two drugs that have been moved forward in the clinic, that is, semagacestat (3) and begacestat (50), displayed 24.9 nM and 17.9 nM potency, respectively, toward the PSEN1–APH1B complex but again low selectivity (6–13-fold) *versus* PSEN2 complexes. The PSEN1 complex selective inhibitor MRK-560 stands out in terms of potency and selectivity displaying subnanomolar potency toward PSEN1–APH1B (0.4 nM), little selectivity *versus* PSEN1–APH1A (4-fold), and >100-fold selectivity *versus* the two PSEN2-containing complexes (PSEN2–APH1A and PSEN2–APH1B). Recent cryo-EM structures of MRK-560 in PSEN1 and PSEN2 confirmed the selectivity of this compound and also provided structural insights into the basis of the selectivity of this drug (18).

We investigated here the structural requirements of a small compound to make it GSI PSEN1–selective. Based on available small-molecule X-ray crystal structures of an MRK-560 analog (42) and another PSEN1 selective inhibitor ELN-475516 (43), we identified a shared “U” conformation between 4-chlorophenyl sulfone/sulfonamide and 2,5-fluorophenyl/pyrazole moieties, which aligned rather well with structures of other PSEN1 selective inhibitors such as ELN-318463, SCH-900229, and SCH-1500022. Apart from the specific “U” conformation and the structural rigidity, the presence of the hydrogen bond donor significantly enhances the potency and the selectivity of the compounds. In contrast, cyclohexyl sulfone analogs of MRK-560 (without a trifluoromethyl sulfonamide moiety and a hydrogen bond donor) displayed significant decrease in potency and selectivity. Similarly, replacing pyrazole with an H-bond donor of ELN-475516 to iso-oxazole or N-Me pyrazole resulted in significant loss of potency (43). PSEN-1 selectivity seems to come with the H-bond donor except for SCH-900229. Based on this analysis, we set out to generate a novel small compound to confirm our assumptions. We shortlisted a [2,2,2] aza-bicyclooctanone scaffold, which provides a good cyclic core that can be substituted with aryl and aryl sulfonamide groups to provide the vital “U” conformation based on computational studies. [2,2,2] Aza-bicyclooctanone sulfonamide (+)-9b (Fig. 1*G*) turned out to be a very potent PSEN1–APH1B complex–selective GSI (IC_50_ of 6 nM), displaying similar selectivity as MRK-560 toward PSEN1–APH1A (4-fold) and very high selectivity (>250-fold) *versus* PSEN2 complexes. This work supports identification of essential structural elements in an inhibitor of γ-secretase complexes that would provide selectivity toward PSEN1 *versus* PSEN2.

Starting from the recent cryo-EM structure of the inhibitor avagacestat bound to human PSEN1–γ-secretase (15), we propose the binding mode of (+)-9b to γ-secretase. Avagacestat displays low nanomolar potency toward PSEN1–APH1B (1.2 nM) but is not highly selective *versus* PSEN2 complexes (41-fold) compared to (+)-9b (>250-fold). The cryo-EM structure shows how and where avagacestat binds in the γ-secretase complex but does not explain the structural motifs and protein interactions required to achieve complex selectivity (51, 52). More recently, the same authors published cryo-EM structures of γ-secretase complexes together with MRK-560 (18). This study confirmed that the binding site of avagacestat and MRK-560 is the same and that two amino acids in this binding site, that is, T281 and L282, are necessary and sufficient for PSEN1 over PSEN2 γ-secretase selectivity.

Our work predicted the compound (+)-9b to bind in the same site in PSEN1 γ-secretase (53). The ligand (+)-9b is seen to make important, mostly hydrophobic, interactions with T421, L422 and L425, and D385, Figure 2*C*, as well as with T281 and L282. Our simulations showed that binding occurred *via* an entrance channel with L172, previously shown to be important in PSEN1 selectivity (38), acting as a gatekeeper. Therefore, we chose L172 for experimental mutagenesis along with more amino acids around the entrance channel. Mutation of T147A, L150A, and Y154A in TM2, of L166A in TM3, of M233A in TM5, and of I387A and F388A in TM7 resulted in decrease of potency of compound (+)-9b. The mutation of the previously mentioned L172 as well as S169 to alanine resulted in an increase in the binding affinity of compound (+)-9b. As depicted in Figure 2, *B* and *C*, L172 is at the entrance of a channel in PSEN1 involving several of the other amino acids studied in this paper and leading to the binding site and amino acids L282 and T281. Alanine mutation of L172 creates a slightly wider entrance to the channel. Compound(+)-9b could reach the binding site in a shorter simulation time only with the L172A variant and not WT (Fig. 2, *A* and *D* in green dots). On the other hand, avagacestat also interacts with L172 during channel entrance but takes longer to reach the binding site for both mutant and WT. This indicates that compound (+)-9b seems to be more affected by this mutation than avagacestat. This could be due to the higher rigidity of (+)-9b explained by the bicyclooctane ring next to the sulfonamide group and that L172 acts as a gatekeeper limiting the entry of the inhibitor. It is noteworthy that the other PSEN1-selective GSIs appear also more conformationally rigid than the nonselective GSIs and the transition state inhibitor analogs. This entrance pore is likely more closed in PSEN2 ƴ-secretase complexes as we find a longer methionine residue at this position, and we speculate that this provides more hindrance than leucine. We believe there are two prerequisites for compounds to achieve the PSEN1 selectivity: first, the rigid conformation of the scaffold so that it can preferentially enter into the wider PSEN1 complex than into the narrower entrance of the PSEN2 complex; secondly, the formation of three H-bond interactions with D385, L282, and L432 of PSEN1 and being in proximity to loop-2 to make favorable interactions.

Previous work has shown that selective inhibition of γ-secretases might be one of the ways forward for further therapeutic development of γ-secretases in AD (19, 21, 54) and cancer (7), other possibilities being direct (53) or indirect (55) modulation or stabilizing (56) of its activity (56, 57). We provide here a structural basis for the first approach and hope that this will stimulate further research into that direction. However, other work from our laboratory has suggested that even further selective inhibition, targeting only the PSEN1–Aph1B complexes, would be particularly beneficial in the prevention of AD (21). While we found some indications in the current work that selectivity between PSEN1–APH1A and PSEN1–APH1B can be achieved, this aim remains rather elusive, and further high-resolution structures including the determination of Aph1B *versus* Aph1A differences would help in this regard.

In conclusion, with the proof that Aβ therapeutics have a place in the fight against AD (51) and the observations in the current and other studies that selective inhibition of γ-secretase (18, 20) is possible and preclinical evidence that this is a more safer way forward (7, 17, 19, 21), further efforts to develop drugs that target specifically PSEN1–APH1B γ-secretase complexes seem an important goal for new therapeutic development. Small compounds that act selectively and more safely could become a cheap and more broadly available alternative than the expensive passive immunization approaches that are currently propagated and have shown success in the fight against AD (51).

## Experimental procedures

### Generation of stable cell lines

Conditional *Psen*1/2 double KO mice were crossed with conditional *Aph1*ABC triple KO mice (29, 52, 58, 59). At embryonic day 7.5, embryos were dissected and dissociated, and cells were plated in the presence of Dulbecco’s modified Eagle’s medium (DMEM)/F12 50% fetal calf serum (FCS) (Invitrogen). Primary MEFs were immortalized by transduction with LargeT antigen. Psen1/2 double KO/Aph1ABC triple KO MEFs were generated by transduction with a Cre-GFP–expressing adenoviral vector, and GFP-positive MEFs were sorted by fluorescence-activated cell sorting analysis. Psen1/2 Aph1ABC-deficient MEFs were maintained in DMEM/f12 10% FCS. To rescue γ-secretase expression, Psen1/2 double KO/Aph1ABC triple KO MEFs were transduced using Murine Stem Cell Virus retroviral vector system (pMSCV) viral vectors (Clontech) containing the human coding sequences of the different PSEN and APH1 homologs and the zeocin selection marker. An Internal Ribosome Entry Site sequence was cloned between the coding sequences for PSEN and APH1 to ensure co-expression of both proteins. Stable transfected cell lines were selected using 500-μg/ml zeocin (Invitrogen). Four different combinations were made: PSEN1 and APH1A_L_, PSEN1 and APH1B, PSEN2 and APH1A_L_, and PSEN2 and APH1B. These cell lines were transduced with pMSCV viral vectors (Clontech) expressing APP-C99-GFP-puromycin. After puromycin selection (5 μg/ml), GFP-positive cells were selected through FACS sorting. For the alanine mutagenesis experiment, pMSCV PSEN1-APH1A viral vectors were generated by using a long PCR-based QuikChange strategy (Stratagene). Stable cell lines without clonal selection were generated for each mutant as described above. All cell lines were regularly tested for the absence of *mycoplasma* and used for maximum 20 passages in culture.

### Testing compounds

The number of plated cells and incubation times were determined in respect to linearity of Aβ peptide secretion, the dynamic range of Aβ peptide quantification in the medium, and sensitivity to dimethylsulfoxide (DMSO). In every plate, avagacestat was tested at 10 μM to determine the noise signal by completely blocking γ-secretase (see also Fig. S2). MEF cells were plated in DMEM F12 supplemented with 10% FCS at 10,000 cells per well in 96-well clear bottom plates in the late afternoon and cultured for 16 h at 37 °C, 5% CO_2_. In the morning of the second day, the medium was replaced with 60 μl of DMEM/F12 supplemented with 2% FCS, and GSI or DMSO (controls) were added. Compounds were tested in serial dilutions with concentrations ranging from 10 mM to 0.1 nM with 3-fold changes. The final concentration of DMSO in all wells was 0.2%. Plates were put in the incubator again at 37 °C, 5% CO_2_. After 8 h, the culture media were collected, and 30 μl was used to measure Aβ40 peptides. The cell viability was assessed using the CellTiter-Glo Luminescent assay (Promega) that measures ATP production. All screens were performed at one site, and reported IC50 values throughout the manuscript are from this site (Beerse) unless otherwise indicated. We however measured five compounds at the Leuven site and noticed that absolute IC50 values were different (Fig. S2). The main goal of the current work is to explore the basis for inhibitor selectivity for different γ-secretases, and selectivity was consistent in the assays at the two sites (Fig. S2).

### Quantification of soluble Aβ peptides using ELISA

Standard 96-well SECTOR plates (MSD) were coated with 1.5 μg/ml anti-Aβ JRFcAβ40/28 capture antibody in a final volume of 50 μl of PBS 0.05% Tween 20. After overnight incubation at 4 °C, the plates were five times rinsed with PBS 0.05% Tween 20 and blocked with 150 μl per well of casein buffer (PBS with 1% casein, pH 7.4) for 4 h at room temperature. Standards (synthetic human Aβ 1–40) were diluted in culture media. Standards and samples were preincubated with JRFAβN/25 (human-specific antibody) labeled with a sulfo-TAG detection antibody in casein buffer for 5′ at room temperature. The blocked assay plate was rinsed five times with PBS 0.05% Tween 20, and the sample and secondary antibody mix was added. After overnight incubation at 4 °C, plates were rinsed with PBS 0.05% Tween 20, and 150 μl of 2 × Rad T buffer (MSD) was added, and plates were read on an MSD Sector S 600 reader without any delay.

### Data calculation

For each MEF cell line, Aβ peptide levels are expressed as percentage of the signal measured for DMSO (control) after subtraction of the signal obtained in the presence of 10-μM avagacestat, which is supposed to completely block all ƴ-secretase activity. Typical signal-to-noise ratios were >10. Z prime scores in all experiments were well above 0.6. GraphPad Prism 7 software (https://www.graphpad.com) was used to generate inhibition fitting curves (four-parameter logistic equation, nonlinear regression) and to determine IC_50_ values and 95% CI.

### Western blot analysis

Fifty micrograms of cleared protein lysate (in 250 mM sucrose, 1 mM EGTA, 5 mM Tris–HCl pH 7.4) supplemented with 1% TX-100 and cOmplete protease inhibitor cocktail (Roche) was loaded in reducing and denaturing conditions on NuPAGE (Thermo Fisher Scientific) gels and subjected to electrophoresis. Following separation, proteins were transferred to a nitrocellulose membrane for Western blotting. Membranes were blocked with 5% nonfat milk Tris-buffered saline, containing 0.1% Tween 20, and incubated with the indicated primary antibodies, washed, and incubated with horseradish peroxidase–conjugated secondary antibodies (Bio-Rad). Blots were developed using the ECL Renaissance kit (PerkinElmer) using a LAS-3000 Imaging System From Fuji. Primary antibodies used in this study were anti-GFP (11814460001, Roche, 1/1000), 9C3 against NCSTN (60), 1/3000, MKAD3.4 against PSEN1 (61) (1/3000) raised in mouse and B82, B78, and B126 against APH1A, APH1B, PSENEN, respectively (62), 1/1000 and PSEN2 (9979, Cell signaling Technology), 1/1000 raised in rabbit.

### Alignment of known Psen1 selective inhibitors

The crystal structure of ELN-47551635 (CCDC 764935) (43) was used to align ELN-318463, ELN-475516, SCH-900229, SCH-1500022, and MRK-560 using the MOE flexible alignment tool from MOE, v2018.01; Chemical Computing Group ULC.

### Chemistry

ELN-318463 (63), MRK-560 (64), MK-0752 (65), ELN-475516 (43), cyclohexyl sulfone (66), and TSAI-1 (67) were synthesized based on the published procedures and were >95% pure as assessed by HPLC. Semagacestat, avagacestat, begacestat, DAPT, RO-4929097, PF-3084014, L-685,458, LY411575, compound 34, and DAPT analog were purchased from commercial providers and were >95% pure as assessed by HPLC.

**Figure 4:**
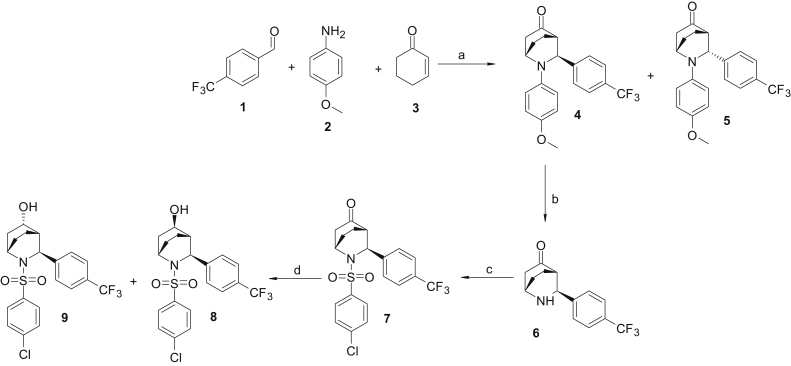
**Synthetic scheme for bicyclootane sulfonamides.** Reagents and conditions *A*, Bi(NO_3_)_3_ .5H_2_O, dimethylformamide, 60 °C, microwave, 2 h, 30 to 60%; *B*, (NH_4_)_2_Ce(NO_3_)_6_, H_2_O:CH_3_CN (1:1), 0 °C, 1 h, 30 to 70%; *C*, R_2_SO_2_Cl, di-isopropyl ethyl amine, CH_2_Cl_2_, 0 °C, 2 to 18 h, 40 to 80%; *D*, NaBH_4_, MeOH, RT, 2 to 18 h, 80 to 95%.

2-Azabicyclo[2.2.2]octane sulfonamides were synthesized as depicted in Figure 4. An equimolar mixture of commercially available 4-(trifluoromethyl)benzaldehyde **1**, *p*-anisidine **2**, 2-cyclohexen-1-one **3**, and catalytic bismuth nitrate pentahydrate (Bi(NO_3_)_3_^.^5H_2_O) in anhydrous dimethylformamide was heated to 60 °C under microwave conditions to generate the 1-*endo* 9 and 1-*exo* 10 diastereomers with good to moderate yields (68). Both diastereomers were separated by flash column chromatography. *p*-Methoxy phenyl deprotection of 4 was achieved with ceric ammonium nitrate at 0 °C in low yields to afford amine 6. *N*-sulfonylation of 7 was achieved using sulfonyl chloride and di-isopropyl ethyl amine in anhydrous dichloromethane to provide ketone sulfonamides 12. Sodium borohydride was employed for the ketone reduction of 12 to afford 3-*exo* 9 and 3-*endo* 8 hydroxy isomers. Enantiomeric separation of 9 was performed using chiral supercritical fluid chromatography techniques to afford (+)-9a and (−)-9b (Fig. 4).

### Ligand entrance simulation using PELE

Schrodinger’s Protein Preparation Wizard was used to add hydrogen atoms, fix structural problems, and generate the L172A variant (protein preparation wizard cite). Rotatable bonds of both ligands were taken to build their rotamer library, and parameters were assigned using Open Force Field 2.0.0 (69). The protein was protonated at neutral pH and parameterized with OPLS2005 (70), and the solvent was treated with an OBC-based implicit solvent (71). Partial charges were calculated using the am1-bcc method implemented in antechamber (72). The adaptive PELE protocol was employed to speed up the entrance of each ligand (47). It consists in applying a set of short PELE simulations (epochs) of several steps, combined with a clustering and spawning strategy to promote the exploration of those regions that have been less explored. A weak bias was also applied to lead the ligand near the binding site, thereby facilitating its entrance.

Each simulation ran on 128 computing cores, and each of them performed 100 epochs of eight PELE steps. A PELE step applies a Monte Carlo step where the ligand is perturbed with a random translation and rotation upon which the system is relaxed through a side chain prediction algorithm and a global minimization. The Metropolis criterion is examined at the end to check if the resulting state can be accepted, following the Boltzmann distribution, or needs to be rejected. The binding site distance was computed taking the distance between the center of mass of the ligand and the carbonyl oxygen of leucine 432 in the binding site, opposite to the proposed entrance channel. The binding energy measures the interaction affinity between protein and ligand, and it is calculated by applying the equation: binding_ energy = total_ energy_ complex − (total_ energy_ protein + total_ energy_ ligand).

### Ligand modeling

Ligand conformers were docked into the cryo-EM using Glide XP. The protein structure with PDB ID 6IDF was prepared using default protein preparation procedures with Maestro software (73). Docking was performed with expanded sampling, and an increased number of solutions per ligand were passed to refinement and to postminimization. The top-ranking docking poses were visually inspected. The docking solution was further studied in explicit cell membrane MD simulations with GROMACS. The complex was embedded in a pre-equilibrated box (9 × 9 × 9 nm containing a lipid bilayer [205 POPC molecules] with an explicit solvent [∼14,000 waters] and 0.15 M concentration of Na^+^ and Cl^−^). The system was energy minimized and subjected to a 5-step MD equilibration (10 + 5 + 2 + 2 + 2 ns) in which constraints in hydrogen atoms, protein loops, and protein and ligand atoms were subsequently relaxed followed by 200 ns of unrestraint MD using a 2-fs time step and constant temperature of 300 K. The AMBER99SD-ILDN force field was used for the protein, the parameters described by Berger *et al.* for lipids, and the general amber force field and HF/6-31G^∗^-derived RESP atomic charges for the ligand. This combination of protein and lipid parameters has been validated for the study of membrane proteins (74).

## Data availability

All study data are included in the article and/or supporting information. The cell lines described in this article are available upon request.

## Supporting information

This article contains supporting information.

## Conflict of interest

L. S., R. N., L. P. B., M. M., V. G., D. T., F. B., M. D., E. F., G. T., P. W. M. R., and H. J. M. G. declare no competing interest. B. D. S. is or has been a consultant for Eli Lilly, Biogen, Janssen Pharmaceutica, Eisai, AbbVie, and other companies. B. D. S is also a scientific founder of Augustine Therapeutics and a scientific founder and stockholder of Muna Therapeutics. B. D. S. holds the Bax-Vanluffelen Chair for AD.
