# Supplementary material for: Selective inhibitors of the PSEN1–gamma-secretase complex
Source: J Biol Chem. 2023 May 9;299(6):104794. doi: 10.1016/j.jbc.2023.104794 (PMC10318456; doi:10.1016/j.jbc.2023.104794)
Supplement: Supporting Information [file mmc1.docx]

**Selective inhibitors of the PSEN1-gamma-secretase complex**

Lutgarde Serneels^1^, Rajeshwar Narlawar^1,2^, Laura Perez Benito^3^, Marti Municoy^4^ , Victor Guallar^5,6^, Dries T’Syen^1^, Maarten Dewilde^1,7^, François Bischoff^2^, Erwin Fraiponts^8^, Gary Tresadern^3^, Peter W.M. Roevens^9^_,_ Harrie J. M. Gijsen^2^ and Bart De Strooper^1,10^

Affiliations:

^1^ VIB Center for Brain and Disease Research and KU Leuven, Department of Neurosciences, Leuven, Belgium

^2^ Discovery Chemistry, Janssen Research & Development, Janssen Pharmaceutica NV, Turnhoutseweg 30, B-2340, Beerse, Belgium

^3^ Computational Chemistry, Janssen Research & Development, Janssen Pharmaceutica NV, Turnhoutseweg 30, B-2340, Beerse, Belgium

^4^ Nostrum Biodiscovery, Jordi Girona 29, Nexus II D128, 08034, Barcelona, Spain

^5^ Barcelona Supercomputing Center, Jordi Girona 29, E-08034 Barcelona, Spain

^6^ ICREA, Passeig Lluís Companys 23, E-08010 Barcelona, Spain

^7^ present address: Laboratory for Therapeutic and Diagnostic Antibodies, Department of Pharmaceutical and Pharmacological Sciences, KU Leuven,B-3000, Leuven, Belgium.

^8^ Charles River Laboratories, Turnhoutseweg 30, 2340 Beerse, Belgium.

^9^ Campus Strategy & Partnerships, Janssen Pharmaceutica NV, Turnhoutseweg 30, B-2340, Beerse, Belgium

^10^ Dementia Research Institute, University College London, London, UK

Corresponding Author: [bart.destrooper@kuleuven.be](mailto:bart.destrooper@kuleuven.be)

**Running title: Selective inhibitors of the PSEN1-γ-secretase complex**

**Supporting Information**

1. Experimental procedures
2. Table S1
3. Supplementary figures
4. Supplementary videos

**Experimental procedures**

**Chemistry**

All reactions were carried out by employing standard chemical techniques under inert atmosphere. Solvents used for extraction, washing, and chromatography were HPLC grade. Unless otherwise noted, all reagents were purchased from Sigma−Aldrich or Acros Organics and were used without further purification. All final compounds were characterized by ^1^H, ^13^C NMR and LC/MS. ^1^H nuclear magnetic resonance spectra were recorded on Bruker spectrometers: 360 MHz and DPX−400 MHz. For the ^1^H spectra, all chemical shifts are reported in part per million (δ) units and are relative to the residual signal at 7.26 and 2.50 ppm for CDCl_3_ and DMSO, respectively. ^13^C chemical shifts are reported as δ values in ppm relative to the residual solvent peak (CDCl3 = 77.16). All final compounds were confirmed to be >95% pure via LCMS methods. All the LC/MS analyses were performed using an Agilent G1956A LC/MS quadrupole coupled to an Agilent 1100 series liquid chromatography (LC) system consisting of a binary pump with degasser, autosampler, thermostated column compartment, and diode array detector. The mass spectrometer (MS) was operated with an atmospheric pressure electrospray ionization (API−ES) source in positive ion mode. The capillary voltage was set to 3000 V and the fragmentor voltage to 70 V, and the quadrupole temperature was maintained at 100 °C. The drying gas flow and temperature values were 12.0 L/min and 350 °C, respectively. Nitrogen was used as the nebulizer gas at a pressure of 35 psi. Data acquisition was performed with Agilent Chemstation software. Analyses were carried out on an YMC pack ODS−AQ C18 column (50 mm long × 4.6 mm I.D.; 3 μm particle size) at 35 °C, with a flow rate of 2.6 mL/min. A gradient elution was performed from 95% (water + 0.1% formic acid)/5% acetonitrile to 5% (water + 0.1% formic acid)/95% acetonitrile in 4.8 min; the resulting composition was held for 1.0 min; from 5% (water + 0.1% formic acid)/95% acetonitrile to 95% (water + 0.1% formic acid)/5% acetonitrile in 0.2 min. The standard injection volume was 2 μL. Acquisition ranges were set to 190−400 nm for the UV−PDA detector and 100−1400 m/z for the MS detector. Optical rotations measurements were carried out on a 341 PerkinElmer polarimeter in the indicated solvents.

***Exo−rac*−(1*S**,3*S**,4*S**)−2−(4−methoxyphenyl)−3−(4−(trifluoromethyl)phenyl)−2−azabicyclo[2.2.2]octan−5−one (4):**

4−trifluoromethyl benzaldehyde (1.57 mL, 11.486 mmol), 2−cyclohexen−1−one (1.112 mL, 11.486 mmol), *p*−anisidine (1.415 g, 11.486 mmol) in anhydrous DMF (15 mL) were placed in a microwave vial and bismuth (III) nitrate pentahydrate (557 mg, 1.149 mmol) was added. The reaction vessel was placed in a microwave reactor and irradiated at 60 °C for 2 h. LCMS indicated the formation of *exo* and *endo* isomers. Reaction mixture was diluted with EtOAc (100 mL) and filtered through celite bed. The celite bed was washed with EtOAc several times and the filtrate was washed with water. The aqueous layer was extracted with EtOAc (3 × 50 mL). Combined organic layer was washed with water, brine, dried over anhydrous MgSO_4_ and rotary evaporated. The residue was purified by flash column chromatography (0 to 7 % EtOAc in *n*−heptane) to afford the desired *exo* isomer

^1^H NMR (400 MHz, CDCl_3_): δ = 7.64 (d, *J =* 8.14 Hz, 2H), 7.52−7.59 (m, 2H), 6.70−6.80 (m, 2H), 6.50−6.56 (m, 2H), 4.76 (d, *J =* 2.20 Hz, 1H), 4.44 (quin, *J =* 2.81 Hz, 1H), 3.71 (s, 3H), 2.78 (td, *J =* 3.08, 18.93 Hz, 1H), 2.67 (q, *J =* 2.86 Hz, 1H), 2.40 (dd, *J =* 1.76, 18.71 Hz, 1H), 2.18−2.30 (m, 1H), 1.85−1.97 (m, 1H), 1.66 (dt, *J =* 2.97, 8.20 Hz, 2H) ppm.

LC−MS m/z: 376 [M + H]^+^

***Rac*−*exo*−(1*S**,3*S**,4*S**)−3−(4−(trifluoromethyl)phenyl)−2−azabicyclo[2.2.2]octan−5−one:**

*rac*−*exo*−(1*S**,3*S**,4*S**)− 3−(4−(trifluoromethyl)phenyl)−2−azabicyclo[2.2.2]octan−5−one (2 g, 5.328 mmol) was dissolved in water (50 mL) and acetonitrile (50 mL) and the resulting mixture was cooled to 0 °C. To this ammonium cerium (IV) nitrate (7.302 g, 13.319 mmol) and H_2_SO_4_ (1M in water, 1 eq) was added and the reaction mixture was stirred for 1 h. After completion (TLC), reaction mixture was basified with 3 M KOH to the pH 9‒10 and the suspension was filtered through dicalite bed. The dicalite bed was washed with EtOAc several times. Layers were separated and the aqueous layer was extracted with EtOAc (3 ×). Combined organic layer was washed with water, brine, dried over anhydrous MgSO_4_, filtered and solvent was evaporated under reduced pressure. The crude residue was purified by flash column chromatography (10−25% EtOAc in *n*‒heptane) to yield the desired amine (0.45 g, yield 31 %) as pale−brown gummy solid.

^1^H NMR (400 MHz, CDCl_3_): δ = 7.60−7.71 (m, 4H), 4.67 (s, 1H), 3.58 (tt, *J =* 1.93, 3.56 Hz, 1H), 2.58−2.67 (m, 1H), 2.43−2.52 (m, 2H), 2.05−2.19 (m, 1H), 1.65−1.80 (m, 2H), 1.51−1.61 (m, 2H) ppm.

LC−MS m/z: 270 [M + H]^+^

***Rac*−*exo*−(1*S**,3*S**,4*S**)−2−((4−chlorophenyl)sulfonyl)−3−(4−(trifluoromethyl)phenyl)−2−azabicyclo[2.2.2]octan−5−one**

(517 mg, 2.451 mmol) was added in one portion to a stirred solution of *rac*−*exo*−(1*S**,3*S**,4*S**)− 3−(4−(trifluoromethyl)phenyl)−2−azabicyclo[2.2.2]octan−5−one (550 mg, 2.043 mmol) and *N*,*N*‒diisopropylethylamine (1.408 mL, 8.17 mmol) in anhydrous dichloromethane (10 mL) at 0 °C. The resulting mixture reaction was warmed to ambient temperature continued stirring for 18 h. After completion (TLC and LCMS), reaction mixture was diluted with dichloromethane (40 mL) and washed with water. The aqueous layer was extracted with dichloromethane (2 ×). Combined organic layer was washed with water, brine, dried over anhydrous MgSO_4_ and solvent was evaporated under reduced pressure. The residue obtained was purified by flash column chromatography (0‒20% ethyl acetate in *n*‒heptane) to afford the titled compound (650 mg, yield 71 %) as colorless solid.

^1^H NMR (400 MHz, CDCl_3_): δ = 7.64−7.69 (m, 2H), 7.61 (d, *J =* 8.36 Hz, 2H), 7.40−7.48 (m, 4H), 5.11−5.25 (m, 1H), 4.44−4.59 (m, 1H), 2.55 (q, *J =* 2.86 Hz, 1H), 2.37−2.47 (m, 1H), 2.21−2.34 (m, 2H), 1.79−1.93 (m, 1H), 1.67−1.78 (m, 1H), 1.56−1.66 (m, 1H) ppm.

LC−MS m/z: 441 [M − H]^−^, 461 [M + NH_4_]^+^

**Synthesis of Compound 8 and 9:**

To a stirred solution of *rac*−*exo*−(1*S**,3*S**,4*S**) −2−((4−chlorophenyl)sulfonyl)−3−(4−(trifluoromethyl)phenyl)−2−azabicyclo[2.2.2]octan−5−one ( 255 mg, 0.574 mmol) in MeOH (10 mL), NaBH_4_ (22 mg, 0.574 mmol) was added in one portion and the resulting mixture was stirred at ambient temperature for 1 h. Upon completion (TLC), solvent was evaporated in vacuo, the residue was diluted with water and acidified with 1N HCl to pH = 4. The resulting mixture was extracted with EtOAc (3 × 25 mL). Combined organic phase was washed with water, brine, dried over anhydrous MgSO_4_ and concentrated in vacuo. The residue was purified preparative HPLC to afford the desired 3−*endo* and 3−*exo* alcohols.

***Rac*−*exo*− *exo*− (1*S**,3*S**,4*S*5R**)−2−((4−chlorophenyl)sulfonyl)−3−(4−(trifluoromethyl)phenyl)−2−azabicyclo[2.2.2]octan−5−ol (8):**

100 mg (39 % yield), colorless solid.

^1^H NMR (400 MHz, DMSO−D_6_) δ = 7.86 (d, *J =* 8.58 Hz, 2H), 7.69 (s, 4H), 7.61−7.66 (m, 2H), 4.83−4.90 (m, 2H), 3.89 (br d, *J =* 1.76 Hz, 1H), 3.58−3.65 (m, 1H), 1.79−1.90 (m, 2H), 1.56−1.72 (m, 3H), 1.08 (dd, *J =* 4.18, 13.86 Hz, 1H), 0.92 (br t, *J =* 10.78 Hz, 1H) ppm.

^13^C NMR (101 MHz, DMSO−D_6_) δ = 146.8 (s), 138.1 (s), 137.9 (s), 129.5 (s), 129.3 (s), 127.4 (s), 127.0 (s), 126.9 (s), 124.6 − 124.9 (m), 66.1 (s), 60.5 (s), 47.7 (s), 40.7 (s), 34.7 (s), 27.0 (s), 10.8 (s) ppm.

LC−MS m/z: 444 [M − H]^−^, 463 [M + NH_4_]^+^

***Rac*−*exo*−*endo*−(1*S**,3*S**,4*S*5S**)−2−((4−chlorophenyl)sulfonyl)−3−(4−(trifluoromethyl)phenyl)−2−azabicyclo[2.2.2]octan−5−ol (9):**

125 mg (48 % yield), colorless solid.

^1^H NMR (400 MHz, DMSO−D_6_) δ = 7.78 (d, *J =* 8.80 Hz, 2H), 7.70 (d, *J =* 8.14 Hz, 2H), 7.65 (d, *J =* 8.80 Hz, 2H), 7.54 (d, *J =* 8.14 Hz, 2H), 5.15 (s, 1H), 4.81 (d, *J =* 2.20 Hz, 1H), 3.94 (br s, 1H), 3.75−3.86 (m, 1H), 1.84−1.98 (m, 1H), 1.76−1.84 (m, 1H), 1.70 (ddd, *J =* 1.54, 9.68, 14.08 Hz, 1H), 1.44−1.57 (m, 1H), 1.21−1.32 (m, 1H), 1.12−1.21 (m, 1H), 1.04−1.12 (m, 1H) ppm.

^13^C NMR (101 MHz, DMSO−D_6_) δ = 147.5 (s), 138.2 (s), 137.8 (s), 129.4 (s), 129.3 (s), 127.2 (s), 126.9 (s), 125.7 (s), 124.8 (q, *J =* 3.3 Hz), 64.6 (s), 55.2 (s), 47.4 (s), 41.2 (s), 34.5 (s), 25.9 (s), 15.4 (s) ppm.

LC−MS m/z: 444 [M − H]^−^, 463 [M + NH_4_]^+^

**Table S1: Optical rotation**

| **Compound** | Specific rotation | conditions |
| --- | --- | --- |
| (+) **9b** | +196 | 589 nm, c = 0.175 w/v %, DMF, 20 ºC |
| (−) **9a** | −162.5 | 589 nm, c = 0.16 w/v %, DMF, 20 ºC |

**Supplementary figures**

**Fig. S1**


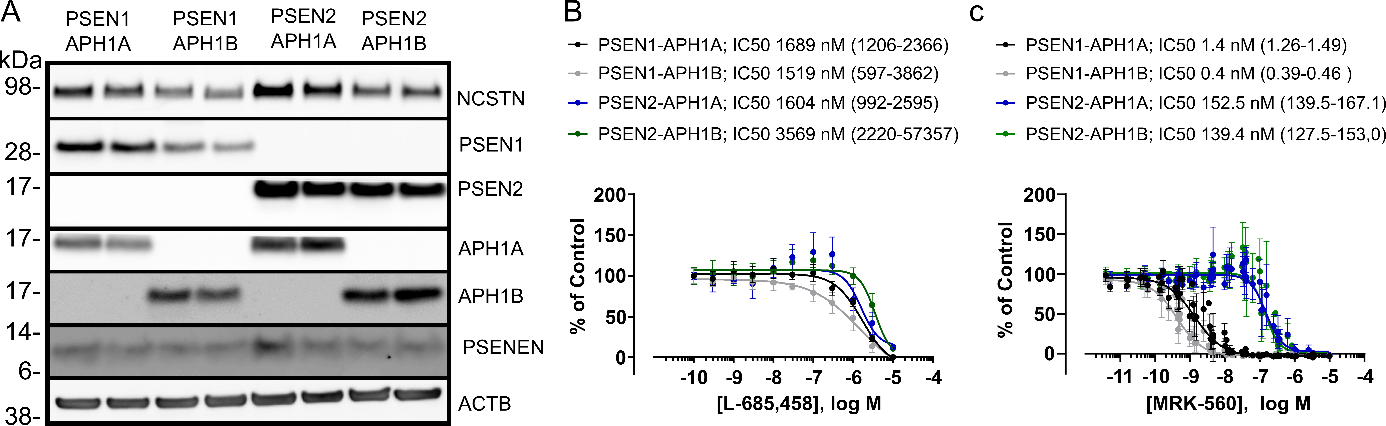


**Fig. S1. Cell based screening assay** **(A)** Western blot analysis of γ-secretase subunits in MEFs deficient for endogenous Psen and Aph1 subunits and rescued with human PSEN1 or PSEN2 and with human APH1A or APH1B. PSEN2 CTF, APH1A and APH1B proteins are running at the same position. Therefore, the same sample lysates were loaded on three different gels and blotted on nitrocellulose membranes. Blots were cut as indicated and stained with the indicated antibodies. Two independent clones were generated of which one was used throughout the study. **(B)(C)** Dose-dependent effects of the transition state inhibitor L-685,458 (B) and PSEN1 selective inhibitor MRK-560 (C) on the production of Aβ_40_-peptides. The data represent ± SD of > 6 experiments, 95% CI are given between brackets. GraphPad Prism 7 software was used to generate inhibition fitting curves (four-parameter logistic equation, non-linear regression) to determine IC_50_ and 95 % CI values.

**Fig. S2**


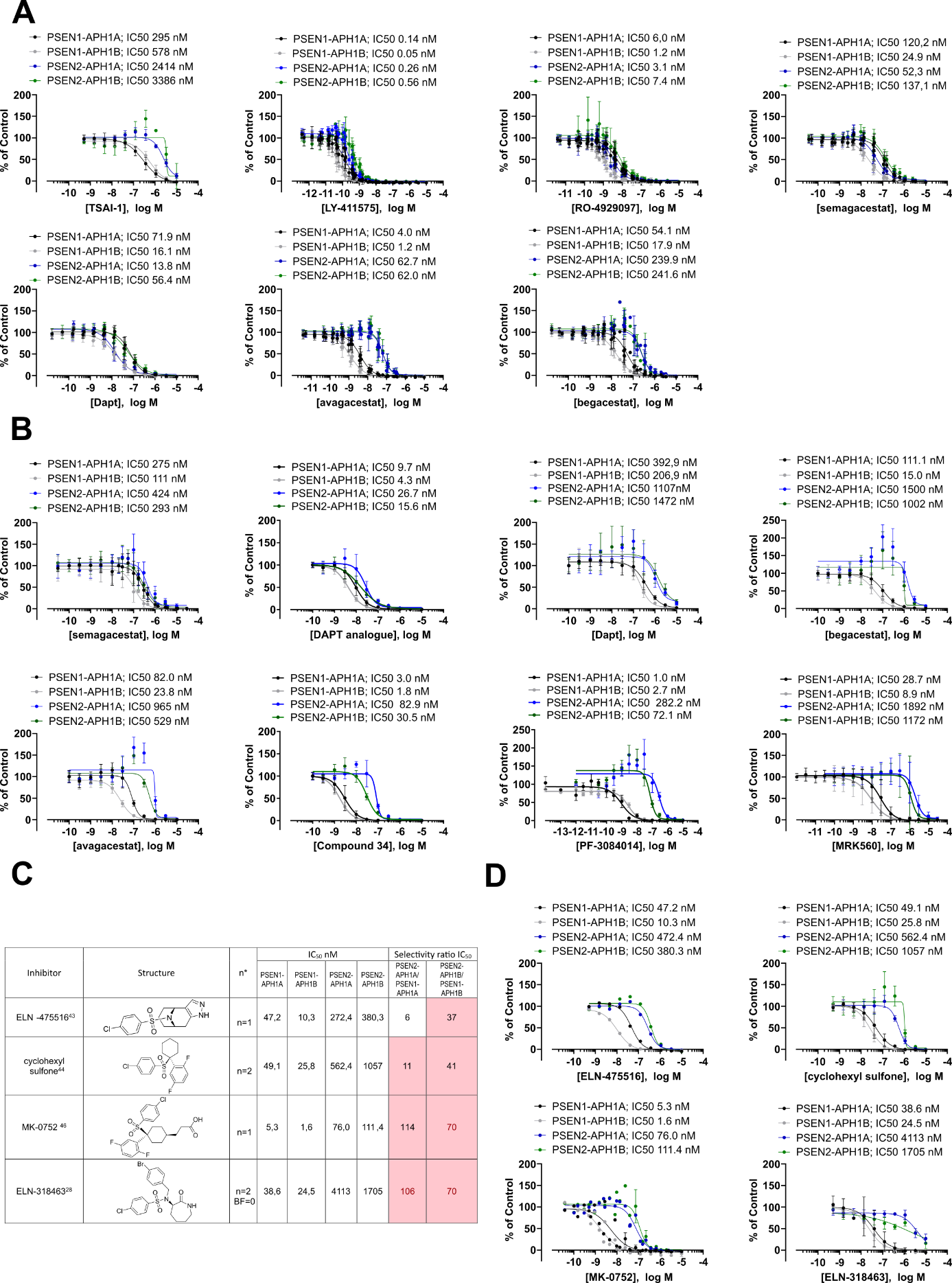


**Fig. S2.** **Dose response curves of data represented throughout the manuscript**. The data shown are means ± SD, number of assays indicated as (N). GraphPad Prism 7 software was used to generate inhibition fitting curves (four-parameter logistic equation, non-linear regression) and to determine IC50 values. Experiments were performed at two sites either Janssen **(A)** or KU Leuven **(B)**. Five compounds (semagacestat, dapt, begacestat, dapt and MRK-560) were tested at both sites. We notice variability in absolute values of IC50 values but importantly the assay shows consistency in γ-secretase complex selectivity. **(C)** and **(D)** show the data discussed throughout the text for compounds that were tested only once or twice during the study but were used to rationalize the design of new compounds. BF indicates “bottom fit” to zero option which was used to determine IC50 for this compound.

**Fig. S3**

**A**


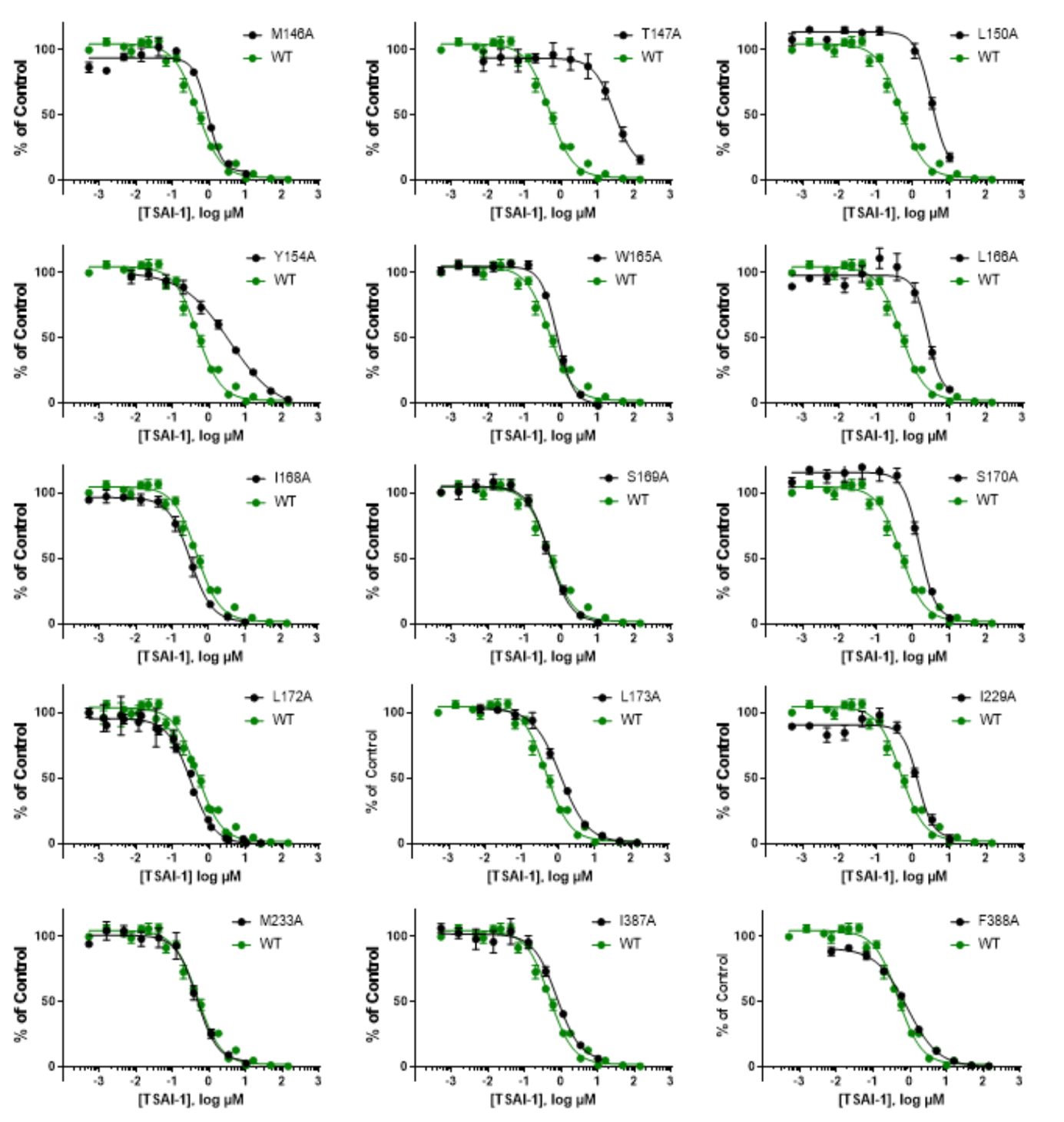


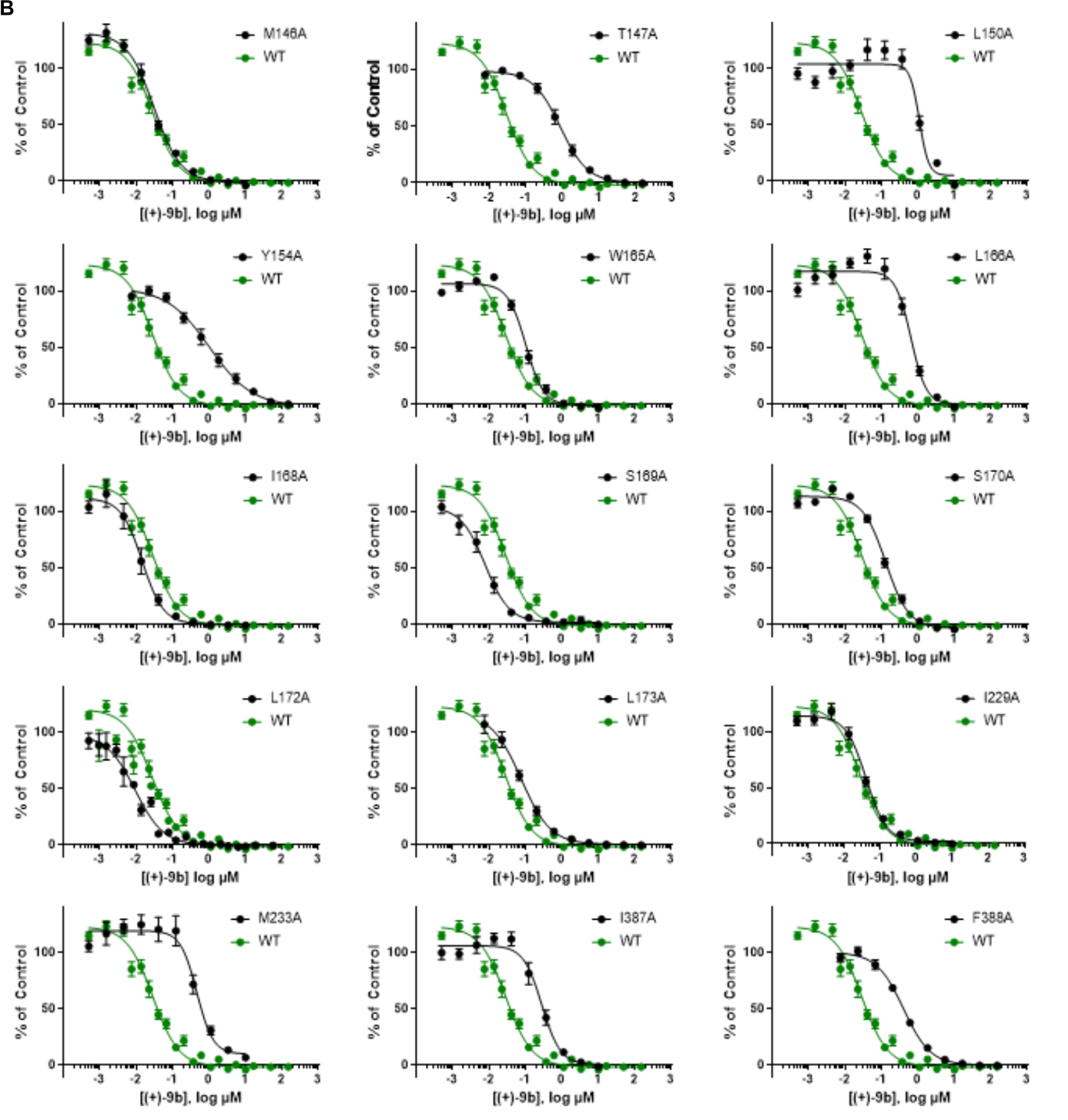


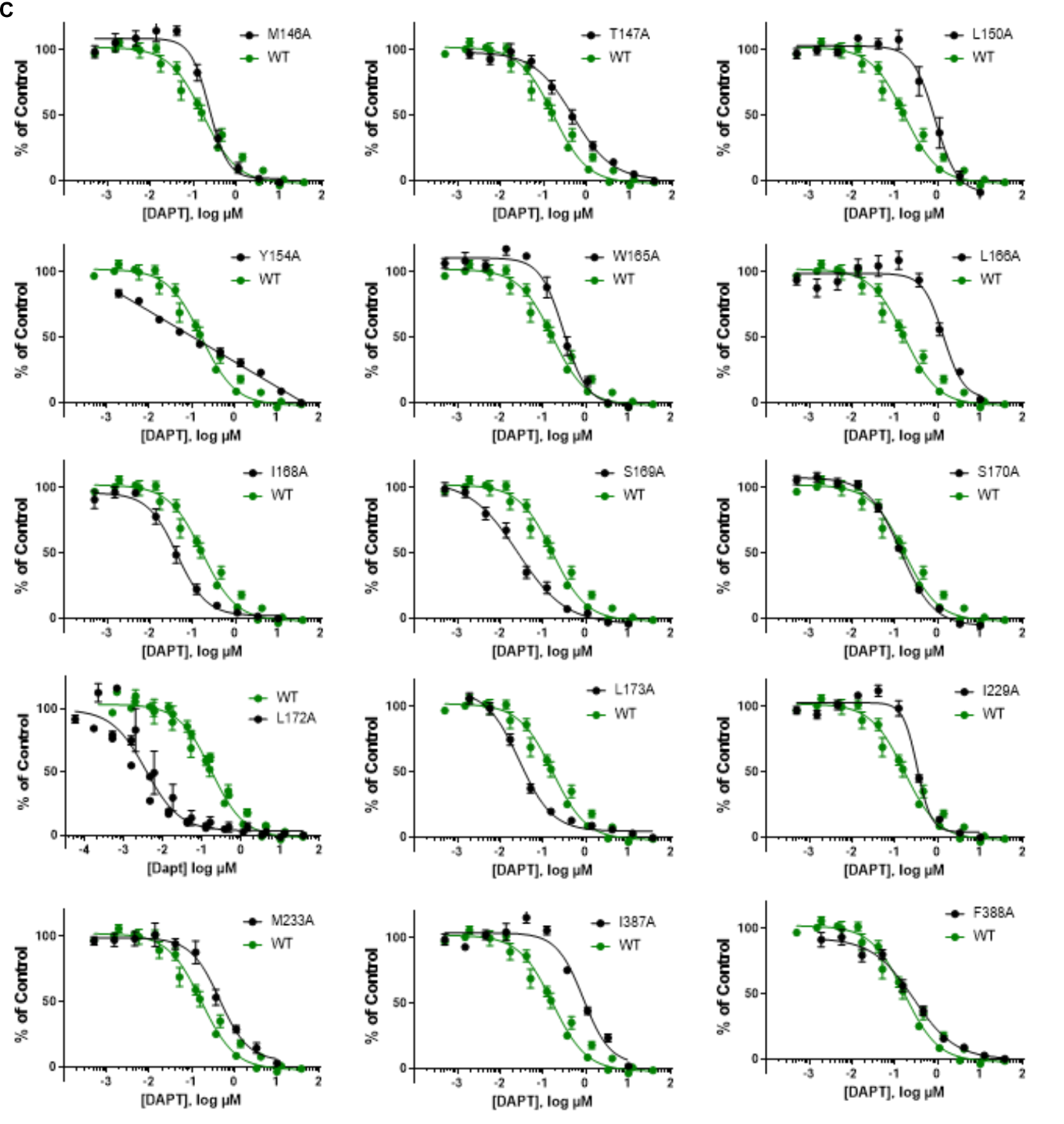


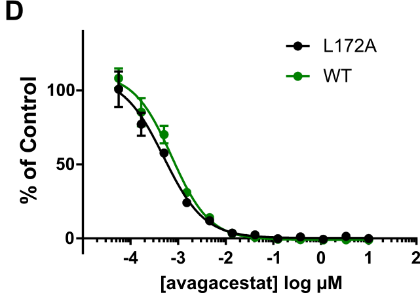


**Fig. S3**. **Dose response curves of data represented in Fig. 2**. The data shown are means ± SD of > 4 experiments. GraphPad Prism 7 software was used to generate inhibition fitting curves (four-parameter logistic equation, non-linear regression) and to determine IC50 values. GSIs used **(A)** TSAI-1, (**B)** (+)-**9b**, **(C)** DAPT and **(D)** avagacestat

**Supplementary videos**

**Video S1**

**Video S1.** Entrance of compound (+)-9b, represented with orange sticks, to native PSEN1 obtained with the out→in protocol of the PELE Platform. Residues along the entrance channel and the binding site are represented with gray sticks. In red, the native leucine at position 172. In green, residue 432 taken as reference to lead the ligand towards the binding site.

**Video S2**

**Video S2**. Entrance of compound (+)-9b, represented with orange sticks, to the L172A variant of PSEN1 obtained with the out→in protocol of the PELE Platform. Residues along the entrance channel and the binding site are represented with gray sticks. In red, the mutated alanine at position 172. In green, residue 432 taken as reference to lead the ligand towards the binding site.
